# Molecular effects of lapatinib in patients with HER2 positive ductal carcinoma in situ

**DOI:** 10.1186/bcr3695

**Published:** 2014-09-04

**Authors:** Laura G Estévez, Ana Suarez-Gauthier, Elena García, Cristina Miró, Isabel Calvo, María Fernández-Abad, Mercedes Herrero, Manuel Marcos, Cristina Márquez, Fernando Lopez Ríos, Sofía Perea, Manuel Hidalgo

**Affiliations:** 1 Breast Cancer Program, Centro Integral Oncológico Clara Campal, C/Oña 10, Madrid, 28050 Spain; 2 Pathology Department, Hospital Universitario Fundación Alcorcón, Madrid, 28922 Spain; 3 Clinical Research Program, Spanish National Cancer Research Center, Madrid, 28029 Spain

## Abstract

**Introduction:**

Human epidermal growth factor receptor 2 (HER2) amplification is frequent in ductal carcinoma in situ (DCIS) of the breast and is associated with poorly differentiated tumors and adverse prognosis features. This study aimed to determine the molecular effects of the HER2 inhibitor lapatinib in patients with HER2 positive DCIS.

**Methods:**

Patients with HER2 positive DCIS received 1,500 mg daily of lapatinib for four consecutive weeks prior to surgical resection. Magnetic resonance imaging (MRI) was used to determine changes in tumor volume. The molecular effects of lapatinib on HER2 signaling (PI3K/AKT and RAS/MAPK pathways), cell proliferation (Ki67 and p27) and apoptosis (TUNEL) were determined in pre and post-lapatinib treatment samples.

**Results:**

A total of 20 patients were included. Lapatinib was well tolerated with only minor and transient side effects. The agent effectively modulated HER2 signaling decreasing significantly pHER2 and pERK1 expression, together with a decrease in tumor size evaluated by MRI. There was no evidence of changes in Ki67.

**Conclusions:**

Four weeks of neoadjuvant lapatinib in patients with HER2-positive DCIS resulted in inhibition of HER2 and RAS/MAPK signaling pathway.

**Trial registration:**

2008-004492-21 (Registered June 25th 2008).

**Electronic supplementary material:**

The online version of this article (doi:10.1186/bcr3695) contains supplementary material, which is available to authorized users.

## Introduction

Data from the National Cancer Database estimate that 12 to 15% of newly diagnosed breast cancer in the US today is ductal carcinoma *in situ* (DCIS) [1]. DCIS is defined as a malignant proliferation of ductal cells of the breast that does not invade through the basal membrane. DCIS rarely presents as a palpable mass in the breast but is associated with microcalcifications seen in mammography. The broad implementation of screening programs has resulted in an increase from 5 to 25% in the number of patients diagnosed with this disease [2, 3].

Conventional management of DCIS includes surgical resection by either mastectomy or breast-conserving surgery and radiation therapy, depending on the extent of the disease [4, 5]. Bilateral breast magnetic resonance imaging (MRI) is being used preoperatively with increasing frequency in women with DCIS, where it has shown high sensitivity for detecting high-grade lesions [6]. Patients with hormone receptor-positive tumors benefit from adjuvant treatment with tamoxifen that decreases local and contralateral tumor failure [2–5]. Despite its excellent prognosis, in up to 10% of patients, DCIS will recur, often with invasive carcinoma [7].

One of the salient features of DCIS is the high expression of human epidermal growth factor receptor 2 (HER2). Although globally 20 to 30% of patients with invasive breast cancer express HER2, the expression is as high as 60 to 70% in patients with high-grade/comedo-type DCIS [8]. Expression of HER2 is associated with high proliferative grade, comedo-necrosis, and p53 mutation, and is inversely associated with the expression of hormone receptors [9, 10].

HER2 belongs to a receptor family that includes HER1, HER2, HER3, and HER4, respectively. These molecules are transmembrane tyrosine kinase receptors with partial homology that regulate cell growth and survival, as well as adhesion, migration, differentiation, and other cellular responses. HER2 are the preferred dimerization partners for the other HER family members [11]. When HER2 induce dimerization with HER3, the phosphorylated (activated) tyrosine residues on the intracellular domain of HER2 activate the lipid kinase phosphoinositide 3-kinase (PI3K), which results in activation of the enzyme AKT transforming factor (AKT) promoting cell survival [11]. In contrast, when HER1 is the chosen partner for dimerization, the complex HER1-HER2 preferentially activate the rat sarcoma (RAS)/rapid accelerated fibrosarcoma (RAF)/mitogen-activated protein kinase (MAPK) cascades promoting cell proliferation [12]. HER2 is a validated therapeutic target in invasive breast cancer, leading to the hypothesis that inhibition of HER2 through anti-HER2 therapy could be beneficial for patients with DCIS.

Lapatinib, a reversible dual-kinase inhibitor of epidermal growth factor receptor (EGFR) and HER2, has activity in HER2-overexpressing breast cancer and is approved in combination with capecitabine for the treatment of patients with metastatic disease [13]. In early pharmacodynamic clinical studies, administration of lapatinib to patients with advanced breast cancer resulted in inhibition of HER2 signaling and induction of apoptosis [14–18]. At the recommended dose, the drug is well-tolerated with skin rash and diarrhea as the predominant toxicities. In rare cases, there were liver and cardiac adverse events [19].

The central hypothesis of the present study is that lapatinib inhibits HER2 signaling in DCIS patients resulting in cell proliferation arrest and apoptosis. The primary objective of the trial was to determine the pharmacodynamic effects of a 4-week course of lapatinib administered preoperatively to patients with HER2-positive DCIS measuring protein expression of phospho extracellular-regulated kinase (pERK), phosphor-AKT (pAkt) and proliferative/apoptosis markers at baseline and post treatment. Secondary analysis included assessment of tumor response by MRI and the correlation with molecular biomarkers.

## Methods

### Study design

This study was a prospective, open-label, phase II trial, conducted at the Centro Integral Oncológico Clara Campal, Hospital Universitario Madrid, between December 2009 and June 2010. Patients were required to give written informed consent before inclusion in the study. The trial was conducted following the guidelines of the local Ethical Review Board and the Spanish Ministry of Health, and in accordance with Good Clinical Practices and the tenets of the Declaration of Helsinki. The protocol was approved by the Clinical Research Ethic Committee of Hospital Universitario Madrid Norte Sanchinarro (Madrid, Spain).

### Eligibility criteria

Female patients with pathological diagnoses of HER2-positive DCIS, according to American Society of Clinical Oncology/College of American Pathologists (ASCO/CAP) guidelines [20], scheduled to undergo surgery by either lumpectomy or mastectomy were eligible. Other eligibility criteria included age ≥18 years, Eastern Cooperative Oncology Group (ECOG) performance status 0 to 2; normal hematological (absolute neutrophil count ≥1.5 cells ×10^9^/L, platelets ≥75 cells ×10^9^/L, and hemoglobin ≥9 g/dl), liver (bilirrubin ≤ 1.25 × upper limit of normal (ULN), aspartate transaminase (AST) and alanine transaminase (ALT) ≤2.5 × ULN and alkaline phosphatase ≤2.5 ×ULN) and renal (creatinine ≤2.0 mg/dL or creatinine clearance >40 ml/min); and normal left ventricular ejection fraction. Lactating and pregnant women were excluded. Other exclusion criteria included previous treatment with lapatinib, contralateral breast mass, presence or suspicion of invasive carcinoma, axillary lymph nodes, malabsorption syndrome that could interfere with lapatinib exposure, any other serious cardiac, neurological or psychiatric disorder that in the investigator's opinion could compromise treatment safety and compliance, and concomitant use of CYP3A4 interacting agents. Women of fertile age were required to use appropriate contraception methods.

### Treatment administration

Patients received lapatinib as single agent administered orally for 28 days (4 weeks) at a dose of 1,500 mg per day divided into six 250-mg capsules, following the package insert recommendations. Missed doses were not made up and no dose reductions were allowed. Treatment compliance was recorded in a patient diary. Patients who for whatever reason, received less than 80% of the prescribed dose were replaced.

### Study procedures and follow up

Patients with histologically confirmed HER2-positive DCIS with measurable residual microcalcifications (1 cm) on mammography after initial diagnostic biopsy were counseled about the study. The first MRI of the breast was also performed after the diagnostic biopsy procedure. Written informed consent was obtained from all patients before study inclusion. Screening studies, including safety laboratory measurements and assessment of left ventricular ejection fraction (LVEF) were performed. Patients were visited on a weekly basis while on medication to monitor treatment compliance and toxicity. Physical examination, hematology and blood chemistry analysis were performed weekly. Treatment-related toxicities were recorded according to the National Cancer Institute (NCI) Common Toxicity Criteria (CTC) version 3 criteria and managed as per-package insert. The second breast MRI was performed on day 28 following the end of lapatinib treatment. Patients were schedule for surgery 3 to 4 weeks (range 1 to 4) after treatment completion.

### MRI technique and interpretation

Imaging studies (mammography and breast MRI) were both performed prior to biopsy procedure and definitive surgery, to permit an unbiased interpretation. Mammography and MRI scans were read and scored independently by one expert breast radiologist. All MRI examinations were performed on a commercially available 3-T system (MR Systems Achieva, Version 2.6.3.6; Philips Electronics 2010). Images were evaluated for areas of abnormal enhancement in the breast using previously described interpretation criteria [21]. Response assessment was defined according to response evaluation criteria in solid tumors (RECIST) [22].

### Tissue processing and biological studies

Biological markers were measured before and after lapatinib treatment in order to evaluate treatment-induced changes. A fresh core needle-biopsy was obtained from each patient prior to lapatinib treatment. Tissue samples were immediately fixed in formalin after the extraction in the radiological room and embedded in paraffin (formalin-fixed paraffin-embedded, FFPE) for molecular tests. After lapatinib treatment, molecular tests were analyzed in representative tumor areas of the surgical specimen. In order to avoid suboptimal fixation of surgical specimens and resulting problem of loss of immunoreactivity of phosphoproteins in paraffin samples, surgical samples were processed as follows: tissue samples were moved from the operating room to the pathology room as soon as surgeons removed the sample from the patient. The samples were sliced at intervals of 5 mm and placed in large volume of formalin for 18 to 24 hours to allow the maximum penetration of formalin and to obtain the optimum fixation of samples. Median time from collection of samples was 8 minutes (range 1 (cores) to 15 (surgical specimens) minutes).

### Fluorescence *in situ*hybridization (FISH)

To assess *HER2* and *EGFR* amplification, fluorescence *in situ* hybridization (FISH) was performed in all samples using the PathVysion HER2 DNA Probe kit (Vysis) and the EGFR Probe kit (Vysis) with the DAKO Histology FISH Accessory Kit. *HER2* gene amplification was considered when the ratio *HER2*/CEP 17 was ≥2.2. *EGFR* gene amplification was considered when the ratio *EGFR*/CEP 7 was ≥2.

### Immunohistochemistry (IHQ)

Immunohistochemical studies were assessed in FFPE tissue for the following primary antibodies: HER2 (SP3 clone; 1:100 dilution; Spring); pHER2 (6B12 clone; 1/100 dilution; Cell Signaling Technology); EGFR (SPM341 clon; 1:25 dilution; Spring), phosphor-EGFR (pEGFR) (Tyr992) (Polyclonal; 1:25 dilution; Cell Signaling Technology), AKT (11E7 clone; 1:50 dilution; Cell Signaling Technology); pAKT (Ser473)(736E11 clone; 1:50 dilution; Cell Signaling Technology), p44/42 MAPK (ERK 1) (137 F5 clone; 1:200 dilution; Cell Signaling Technology), pp44/42 MAPK (pERK1) (Thr202/Tyr204; E10) (20G11 clone; 1:200 dilution; Cell Signaling Technology), phosphatase and tensin homolog (PTEN) (28H6 clone; 1/100 dilution; Novocastra), p27 (SX53G8 clone; Ventana-Ready to use), and Ki67 (30 to 9 clone; Ventana-Ready to use). All antibodies except p27, Ki67 and HER2 were scored based on intensity from 0 to 3+ and percentage of positive cells to generate a score (*H*-score) that ranges from 0 to 300 by multiplying these two parameters. p27 and Ki67 proliferative index were reported as percentage of positive nuclei in hot-spot areas. HER2 was scored following ASCO/CAP scoring criteria for immunohistochemistry. For biomarkers determined by *H*-score, overexpression was considered for scores ≥150. The percentage of stained nuclei was evaluated independently of the intensity for Ki67 and p27 (cutoff ≥15%).

### Mutational analysis

Real-time polymerase chain reaction (PCR) mutational screening for phosphatidylinositol-4,5-bisphosphate 3-kinase, catalytic subunit alpha (PIK3CA) (exons 9 and 20) was carried out using PCR and direct sequencing following standard protocols [23].

### Apoptosis cell analysis

Terminal deoxynucleotidyl transferase-mediated deoxyuridine triphosphate-biotin nick-end labeling (TUNEL) was performed according to the manufacture instructions (Roche Diagnostics, Indianapolis, IN) [24]. The number of apoptotic cells was determined in each sample.

### Statistical and data analysis

This was a single-arm phase-II study to test the hypothesis that lapatinib inhibits the HER2 pathway and induces apoptosis in patients with HER2-positive DCIS. The primary endpoint of the study was the pharmacodynamic effect of the drug on cell signaling, proliferation and apoptosis. The secondary endpoints included tumor changes on MRI. A sample size of 20 patients was arbitrarily selected for this study. For the analysis of the primary endpoint, each biomarker was evaluated before and after treatment and was summarized using descriptive statistics. A decrease or increase in the expression of each single biomarker was determined as a change in the *H*-score. The differences in this parameter before and after treatment were compared with the paired student *t*-test. The SPSS v.19 statistical program was used for all statistical analyses. A two-tailed value of *P* <0.05 was considered statistically significant.

## Results

### Patient characteristics

A total of 20 patients, whose principal characteristics are listed in Table 1, were enrolled. Most patients had high-grade DCIS of non-comedo-type carcinoma and approximately half (60%) of the subjects had positive hormone receptors.

**Table 1:** P**atients’ baseline characteristics**

Characteristic	Value
**Age, median (range)**	51 (38 to 68)
**Eastern Cooperative Oncology Group performance status, number of patients**	
0	20
**Histological subtype, number of patients**	
Comedo carcinoma	7
Non-comedo carcinoma	13
**Pathological subtype, number of patients**	
Low grade	0
Intermediate grade	4
High grade	16
Without necrosis	4
**Hormone receptor status, number of patients**	
Estrogen receptor-positive	12
Estrogen receptor-negative	7
Progesterone receptor-positive	9
Progesterone receptor-negative	10
Unknown	1

### Treatment compliance, safety and type of surgery performed

Seventeen patients completed the prescribed treatment. One patient received 250 mg daily dose instead of the 1,500 mg dose. In addition, two patients interrupted treatment by mistake (miss treatment on day 3 and on day 10) and unrelated side effect. Overall, the treatment was well-tolerated with no episode of grade-3 toxicity. The most frequent adverse events were skin reaction, with 35% of patients experiencing grade-1 rash, and gastrointestinal toxicity, with 50% of patients experiencing grade-1 to grade-2 diarrhea. The types of surgery performed were: conservative surgery (n = 13, 65%) and mastectomy (n = 7, 35%).

### Biomarkers before lapatinib treatment

All the patients were positive for HER2 gene amplification with a median ratio of 7 (range 4 to 11.8). None of the patients had positive EGFR FISH, therefore, it was not evaluated on definitive surgery. At baseline, 16 patients (94%) had pHER2 overexpression, 8 patients (42%) had pAKT overexpression and 7 patients (37%) had pERK1 cytoplasmatic overexpression. PTEN was not expressed in four patients (21%). Sixteen patients (84%) had Ki67 ≥ 15%, whereas low p27-staining (<15%) was observed in half of tumors. PI3KCA mutations were detected in only one patient (6%).

### Effects on signaling pathways, cell proliferation, and apoptosis

**Figure 1:**
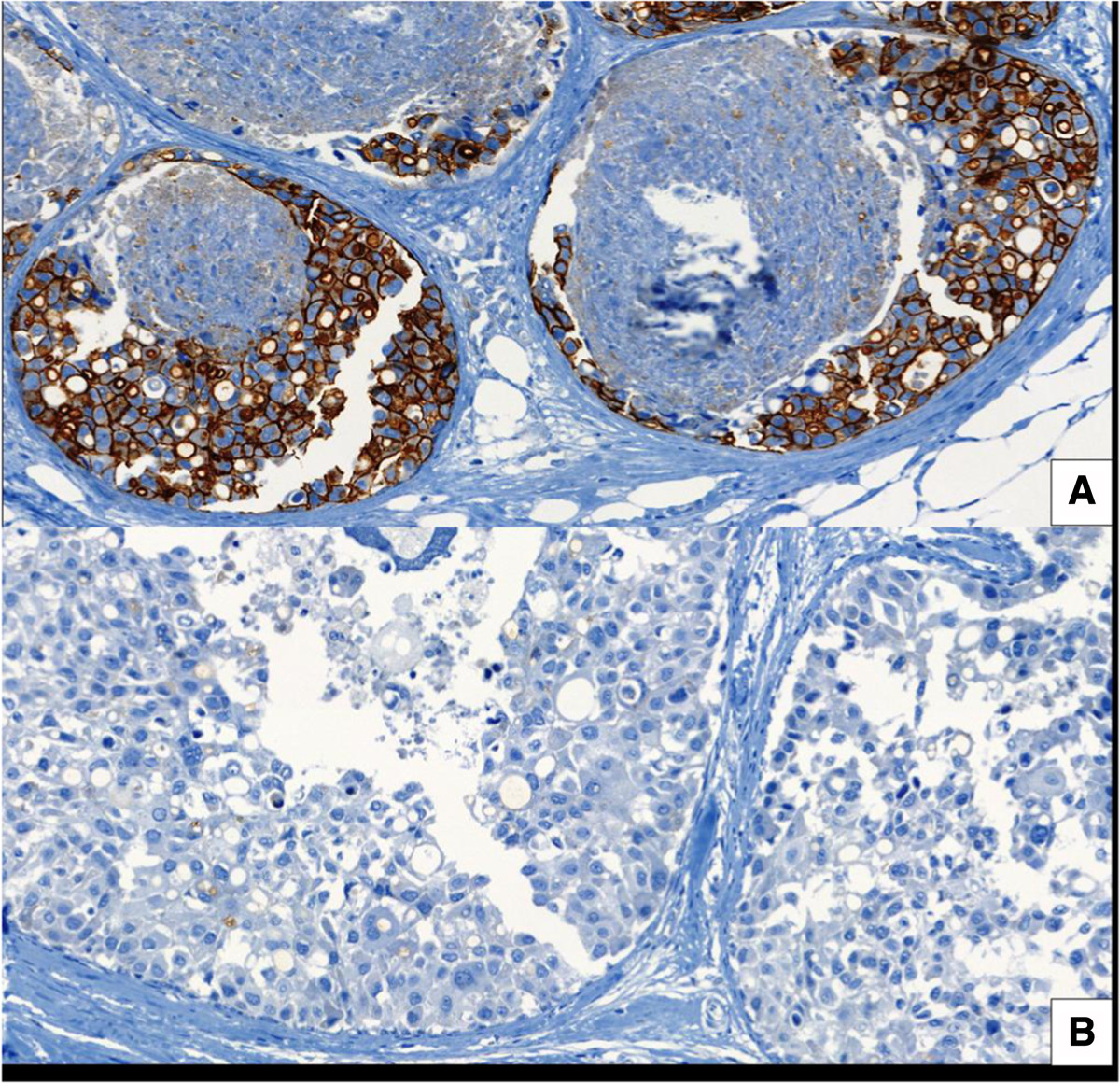
**Phospho human epidermal growth factor receptor 2 (pHER2) protein expression. (A)** Positive expression before lapatinib treatment. **(B)** Negative expression after lapatinib treatment.

**Figure 2:**
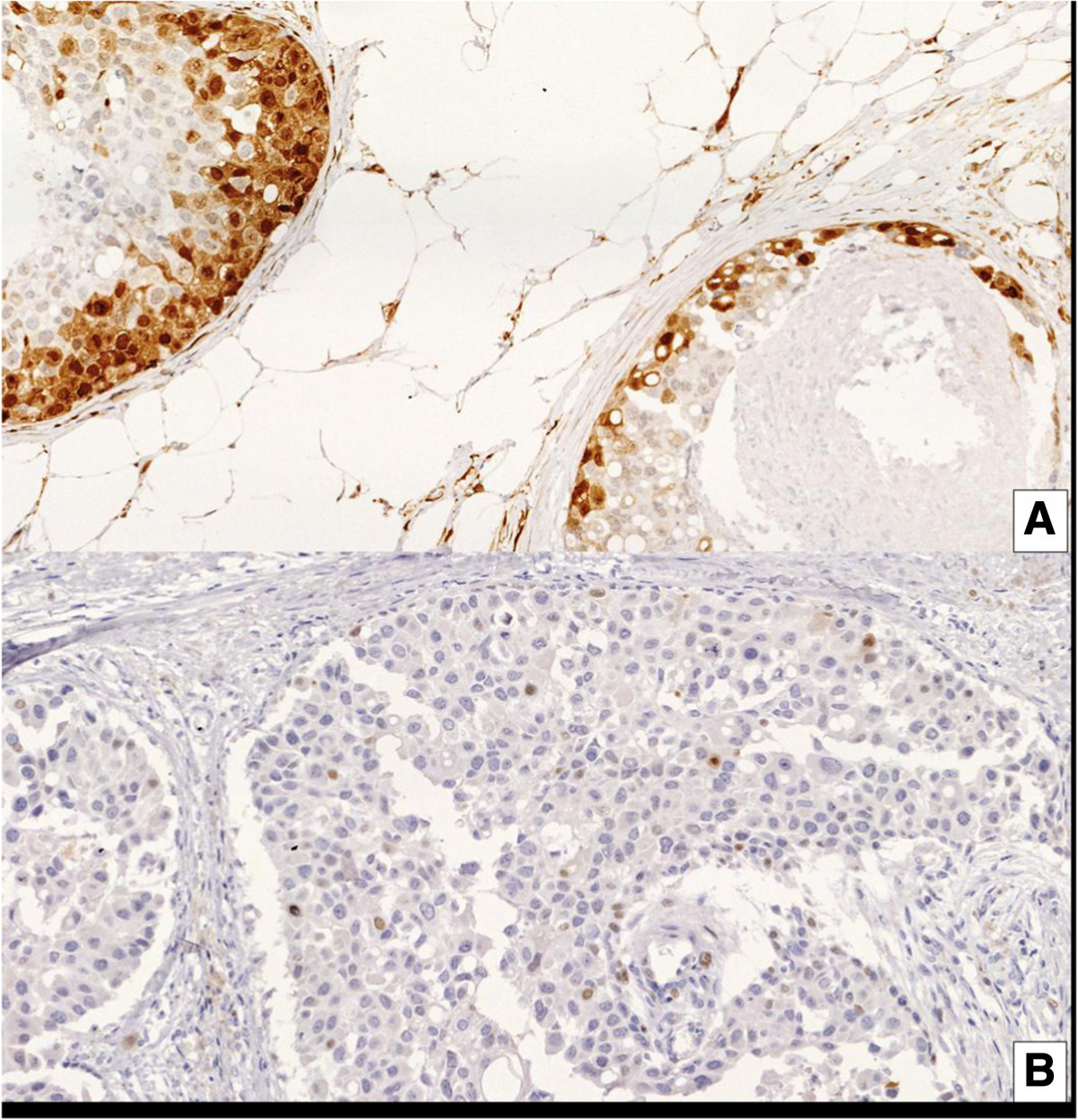
**Phosphor extracellular-regulated kinase 1 (pERK) protein expression. (A)** Positive expression before lapatinib treatment. **(B)** Negative expression after lapatinib treatment.

After 4 weeks of lapatinib treatment surgery was performed in the 20 patients. No evidence of carcinoma *in situ* was noted in two patients after definitive surgery. Biomarkers were evaluated in the 18 remaining patients. Tables 2 and 3 summarize the effects on signaling pathways in each patient individually and in the overall population, respectively. With regard to the HER2 signaling pathway, 13 patients had pre- and post-treatment samples for pHER2 assessment. Overall, there was a statistically significant decrement in pHER2 mean *H*-score from 237.08 pre-treatment to 79.85 post-treatment (*P* <0.001). Of these 13 patients, 12 had pHER2 overexpression as defined in the study (*H*-score ≥150) and in 9 of them there was a reduction of <150 after treatment (Figure 1). Regarding the pERK1, 16 patients had paired samples before and after treatment. In the overall population with matched specimens there was a statistically significant decrease in the mean *H*-score from 117.44 pre-treatment to 45.19 post-treatment (*P* <0.008). pERK1 expression decreased from a *H*-score ≥150 to an *H*-score <150 in five our seven patients (Figure 2). Individually, three patients showed increased expression and in two patients no changes were detected in global matched specimens. Regarding pAKT, in 15 paired samples before and after treatment, no significant differences in the *H*-score (*P* <0.620) were detected. In these matched samples, in four patients with pAKT overexpression at baseline, two had a decrease in the *H*-score below 150 after treatment. Finally, with regard to biomarker correlation, four out of nine patients (44%) with pHER2 reduction (*H*-score ≥150 to *H*-score <150), also presented a decrease in pERK1 expression with almost no changes in pAKT expression. Interestingly, one patient with marked inhibition of pHER2 and pERK1 paradoxically had a increase in pAKT.

**Table 2:** **Changes in** ***H*** **-score in pHER2, pERK1 and pAKT**

Patientnumber	pHER2 expression						pERK1 expression						pAKt expression					
	Pre-lapatinib			Post-lapatinib			Pre-lapatinib			Post-lapatinib			Pre-lapatinib			Post-lapatinib		
	Intensity	Percentage	*H*-score	Intensity	Percentage	*H*-score	Intensity	Percentage	*H*-score	Intensity	Percentage	*H*-score	Intensity	Percentage	*H*-score	Intensity	Percentage	*H*-score
1	N/A	N/A	N/A	2+	90%	180	3+	26%	78	1+	46%	46	1+	5%	5	1+	40%	40
2	3+	91%	273	3+	90%	270	3+	77+	231	2+	76%	152	1+	80%	80	2+	95%	190
3	3+	90%	270	2+	43%	86	2+	80%	160	1+	2%	2	2+	50%	100	3+	3+	285
4	3+	90%	270	1+	77%	77	1+	10%	10	1+	30%	30	2+	95%	190	2+	95%	190
5	3+	90%	270	N/A	N/A	N/A	1+	10%	10	N/A	N/A	N/A	3+	90%	270	N/A	N/A	N/A
6	N/A	N/A	N/A	3+	90%	270	N/A	N/A	N/A	2+	10%	20	N/A	N/A	N/A	1+	95%	95
7	2+	47%	94	1+	5%	5	1+	20%	20	1+	8%	8	2+	90%	180	1+	95%	95
8	3+	90%	270	N/A	N/A	N/A	0	0	0	0	0	0	2+	95%	190	N/A	N/A	N/A
9	3+	90%	270	0	0	0	1+	80%	80	0	0	0	1+	90%	90	1+	90%	90
10	3+	50%	150	0	0	0	1+	90%	90	0	0	0	3+	90%	270	1+	95%	95
11	3+	73%	219	N/A	N/A	N/A	1+	10%	10	N/A	N/A	N/A	3+	90%	270	N/A	N/A	N/A
12	N/A	N/A	N/A	1+	5%	5	0	0	0	0	0	0	0	0	0	0	0	0
13	2+	90%	180	N/A	N/A	N/A	1+	10%	10	N/A	N/A	N/A	3+	90%	270	N/A	N/A	N/A
14	3+	90%	270	1+	10%	10	1+	80%	80	2+	90%	180	0	0	0	1+	95%	95
15	3+	90%	270	0	0	0	2+	95%	190	0	0	0	0	0	0	0	0	0
16	3+	95%	285	3+	90%	270	2+	95%	190	0	0	0	1+	95%	95	1+	95%	95
17	3+	50%	150	2+	50%	100	1+	95%	190	0	0	0	1+	95%	95	1+	95%	95
18	3+	95%	285	0	0	0	2+	55%	110	0	0	0	1+	95%	95	1+	90%	90
19	3+	75%	225	1+	10%	10	3+	70%	210	2+	10%	20	1+	95%	95	1+	95%	95
20	3+	90	270	3+	70%	210	3+	80%	240	3+	95%	285	3+	100%	300	3+	100%	300

Patients 5 and 13 had no residual disease at surgery. pHER2, phospho human epidermal growth factor receptor 2pERK1, phosphor extracellular-regulated kinase 1; N/A, not available.

**Table 3:** **Biomarker analysis in matched specimens**

Biomarker	Number	Mean	SD	*P*-value
**Ki67**				
Pre-lapatinib	16	26.81	12.303	0.051
Post-lapatinib	16	32.44	14.166	
**p27**				
Pre-lapatinib	15	17.67	21.672	0.127
Post-lapatinib	15	8.73	15.050	
**pHER2**				
Pre-lapatinib	13	237.08	63.357	0.000
Post-lapatinib	13	79.85	104.311	
**pERK-1**				
Pre-lapatinib	16	117.44	84.806	0.005
Post-lapatinib	16	45.19	84.673	
**pAKt**				
Pre-lapatinib	15	106.33	93.512	0.620
Post-lapatinib	15	117.00	88.657	

pHER2, phospho human epidermal growth factor receptor 2; pERK1, phosphor extracellular-regulated kinase 1.

**Figure 3:**
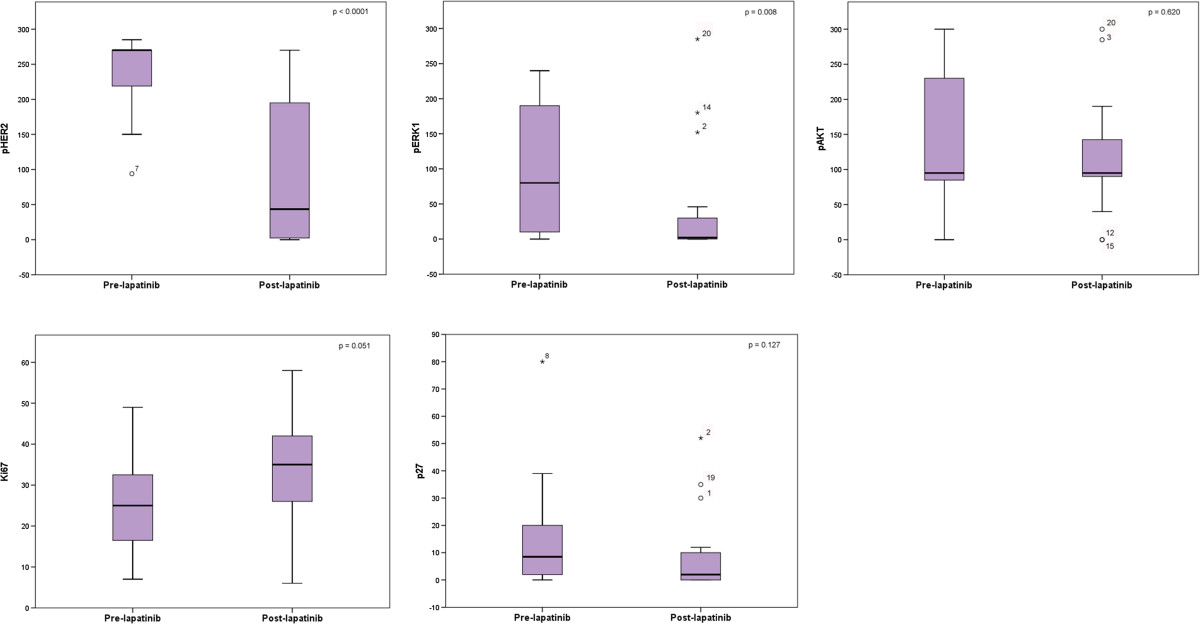
**Changes in biomarkers before and after lapatinib.** pHER2, phospho human epidermal growth factor receptor 2; pERK1, phosphor extracellular-regulated kinase 1.

Apoptosis index through a TUNEL assay was only performed in eight patients due to insufficient specimens and to three of them having an increase in apoptotic levels. The Ki67 index was analyzed before and after treatment in 16 patients with no significant differences in the global population. Likewise, the expression of p27 did not change significantly before and after lapatinib treatment (Table 3). Figure 3 depicts changes in the evaluated tumor biomarkers.

### Treatment response assessed by MRI

**Figure 4:**
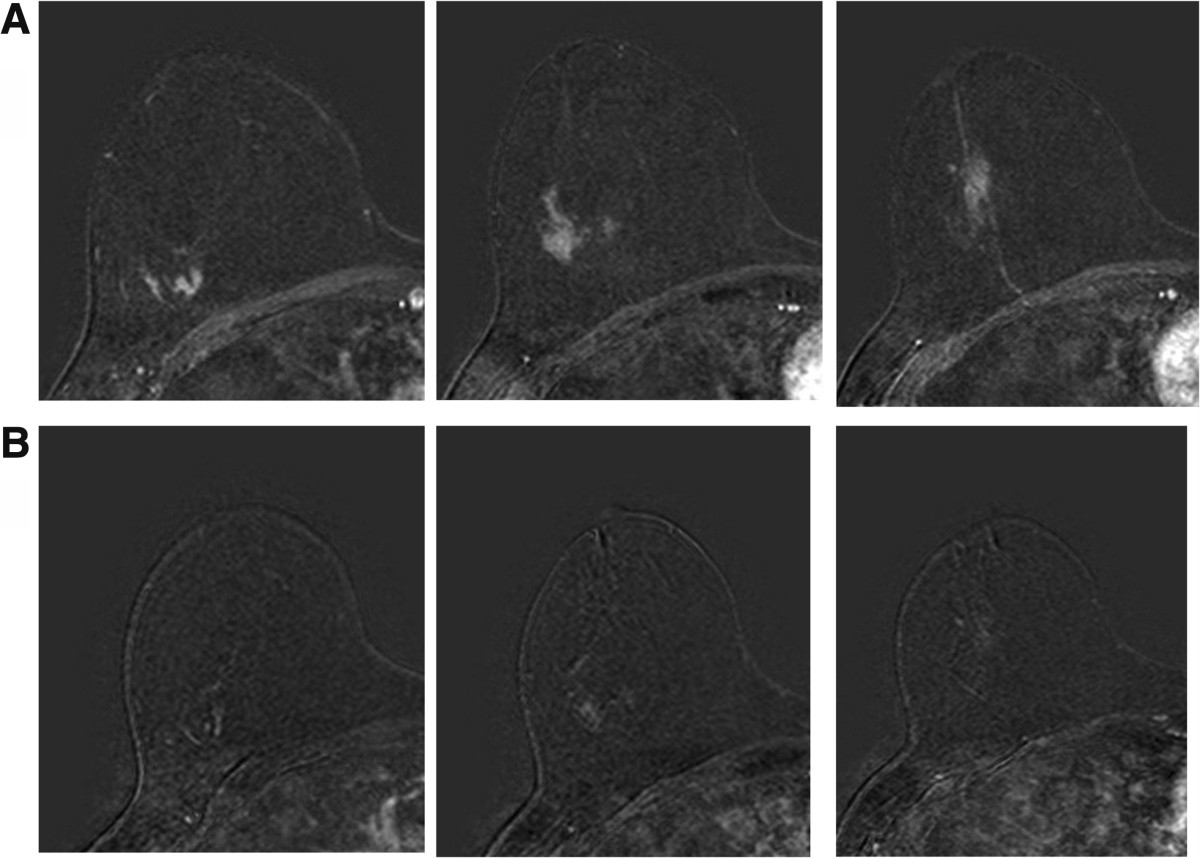
**Treatment response assessed by MRI before (A) and after (B) lapatinib treatment.**

All the patients included in this study underwent MRI before and after lapatinib treatment. MRI findings at the time of diagnosis showed Breast Imaging Reporting and Data System (BIRADS)-2 in eight patients with no evidence of abnormal images. These eight patients presented also with BIRADS-2 in the second MRI performed after lapatinib treatment. The remaining 12 patients had abnormal MRI before lapatinib treatment. The most common abnormality consisted of a non-nodular density with focal, linear, ductal, segmental or diffuse aspect. Of these 12 patients, 9 had a decrease in tumor volume after lapatinib treatment when both MRI studies were compared (Figure 4). In addition, in five out of nine patients (56%) with a decrease in pHER2 expression, a reduction in tumor volume on MRI, or signal intensity, was also observed. The same situation was found in two patients (50%) with reduced pERK1 where a decrease in the signal and tumor size on MRI was also observed (Table 4).

**Table 4:** **Changes in biomarkers and magnetic resonance imaging (MRI)**

Patientnumber	MRI response	pHER2 reduction	pERK1 reduction	MRI-pHER2 correlation	MRI-pERK1 correlation
**1**	No Change*	NA	No	NA	NA
**2**	PR	No	No	No	No
**3**	PR	Yes	Yes	Yes	Yes
**4**	PR	Yes	No	Yes	No
**5**	No change*	NA	NA	NA	NA
**6**	No change*	NA	NA	NA	NA
**7**	No change*	No	No	NA	NA
**8**	PR	No	No	No	No
**9**	PR	Yes	No	Yes	No
**10**	PR	Yes	No	Yes	No
**11**	PR	NA	NA	No	No
**12**	No change*	NA	No	NA	NA
**13**	No change*	NA	NA	NA	NA
**14**	SD	Yes	No	No	No
**15**	No change*	Yes	Yes	NA	NA
**16**	SD	No	No	No	No
**17**	PR	Yes	Yes	Yes	Yes
**18**	PR	Yes	No	Yes	No
**19**	No change*	Yes	Yes	NA	NA
**20**	SD	No	No	No	No

pHER2, phospho human epidermal growth factor receptor 2; pERK1, phosphor extracellular-regulated kinase 1; SD, stable disease (response evaluation criteria in solid tumors (RECIST) criteria); NA, not applicable; PR, partial response (RECIST criteria); no change*, patients with Breast Imaging Reporting and Data System (BIRADS)-2 at diagnosis.

## Discussion

This study is the first to report the use of neoadjuvant lapatinib for patients with HER2-positive DCIS. This pharmacodynamic study aimed to determine the molecular activity of the HER2 inhibitor lapatinib in patients with HER2-positive DCIS. Using a preoperative window of treatment administration for 4 weeks prior to surgical resection, the study showed that the agent is effective in downregulating the HER2 pathway, with slight tendency in the *RAS/MAPK* pathways with virtually no effect against the *PI3K/AKT* pathway. In addition, the agent induced apoptosis as previously seen in invasive breast cancer; however the number of patients was too small to draw any strong conclusions. These molecular effects were associated with significant tumor regression on MRI.

The antitumor effects of HER2 inhibitors require the modulation of key signaling pathways and cell cycle/apoptosis regulatory molecules that mediate the transforming effects of HER2 activation. Recent data in invasive breast cancer support the observation that activation of the *PI3K* pathway or loss of phosphatase and tensin homolog (PTEN) is associated with resistance to trastuzumab [25, 26]. Mechanisms for lapatinib resistance are less well-established. In a recent trial that aimed to evaluate the differential effects of trastuzumab and lapatinib under low and normal PTEN conditions in HER2-overexpressing breast cancer cell lines, lapatinib was effective and decreased both pMAPK and pAKT under low PTEN conditions. In addition, lapatinib showed a significant decrease in Ki67 [27]. As opposed to these findings, the results in the present study showed an increase in Ki67 after lapatinib treatment. It is difficult to explain such results based on well-established scientific evidence reported in the literature as well as on the mechanism of action of lapatinib. Therefore, it is imperative to confirm such results in other patient cohorts that receive the same treatment scheme.

A similar trial with trastuzumab in HER2-positive DCIS was conducted at the MD Anderson Cancer Center in Houston by Kuerer *et al*. [28]. In that study, a single loading dose of 8 mg/kg of trastuzumab was administered to test the biological effects of the agent on DCIS 3 to 4 weeks before surgical resection. In that study, 12 patients received the study drug and 12 patients served as controls with no treatment. Pre- and post-treatment tissues with DCIS were studied for proliferation and apoptosis in patients treated with trastuzumab. The conclusion was that single-dose monotherapy with trastuzumab for patients with HER2-positive DCIS does not result in significant clinical, histologic, proliferative, or apoptotic changes, but results in antibody-dependent cell-mediated cytotoxicity (ADCC)-mediated response through natural killer (NK) cells, and may also induce humoral immunity in a T-cell-dependent manner.

One of the weaknesses of the present study is the lack of a control group. The fact that the present study was performed in only one institution, and not being a multicenter study, prevented the recruitment of a control arm of DCIS untreated patients.

The prognosis of patients with DCIS with conventional surgical and medical treatment is very good and results in cure in the majority of patients. Some patients with extensive disease and aggressive tumors, however, need a mastectomy for full tumor eradication. Interestingly, DCIS has HER2 amplification with higher frequency than invasive tumors and this HER2-positive DCIS is often associated with comedo DCIS which carries twice the risk of local recurrence as compared to non-comedo DCIS [29, 30]. Because of the availability of HER2 inhibitors for cancer treatment, testing whether or not these agents are effective in HER2 DCIS is reasonable. However, given the excellent prognosis of this disease, determining their clinical effect will require a large and lengthy study. In this sense, the ongoing National Surgical Adjuvant Breast Project (NSABP) B-43 phase-III randomized trial in patients with HER2-overexpressing DCIS tests the efficacy of adding trastuzumab to conventional surgical and radiation therapy treatment.

The present study used a preoperative 4-week window between diagnosis and definitive surgery to assess biomarker modulation. Data from the literature have shown that tumor manipulation due to diagnostic procedures such as core biopsies do not induce either molecular changes or changes in biomarker determination in the post-treatment biopsy [31]. However, loss of immunoreactivity of phosphorylated antibodies in paraffin samples may produce significant differences in the results associated with the inappropriate elapsing time of sample fixation. In fact, extreme loss of immunoreactive p-Akt and p-Erk1/2 during routine fixation of primary breast cancer has been reported [32]. However, the present study utilized optimum elapsing time for sample fixation in order to overcome this problem.

*In vitro* studies have shown that blockade of EGFR and HER2 receptors by monoclonal antibodies inhibits cell proliferation. In our study, lapatinib affected HER2 signaling pathways and downregulated the RAS/MAPK cascade throughout moderate reduction in pERK1 protein expression, with no effect in pAKT expression. These biological effects were reflected in signal reduction on MRI. A paradoxical activation of pAKT was observed in one patient with inactivation of pHER2 and pERK1. This negative feedback-loop has been recently described as resulting from HER3 hyperactivation, suggesting that dual inhibition of both pathways should be the most adequate and effective therapy [33].

Several important issues, including the optimal biomarker, methods to determine such biomarkers, and scores to discriminate between positive and negative biomarkers, remain unknown. Unlike for HER2 testing, for which there is currently an international consensus for positive or negative status, there is no consensus on other biomarkers in the *HER2* signaling pathway that are indicative of pathway inhibition [34]. The present study evaluates the downregulation of biomarkers in the *RAS/MAPK* and *PI3K/AKT* pathways by IHC before and after lapatinib administration in order to determine the mechanism of action of this drug in DCIS. The result of this study is that lapatinib results in inhibition of *HER* and *RAS/MAPK* pathways with no effect in *PI3K/AKT* as well as the Ki67 proliferative index. However, there may be other elements in the *HER* signaling pathway that are more relevant to the actions of lapatinib. In addition, we have arbitrarily selected an *H*-score cutoff point to determine positive and negative expression, which may not necessarily be biologically relevant. A study correlating biomarker modulation with pathological response may be more informative in that regard. The fact that over half of the patients with pHER2 downregulation had a reduction in tumor size measured by MRI might indicate a possible activity of lapatinib in DCIS. Indeed, two patients had no residual tumor at the time of surgery in this study. While we cannot rule out that these small tumors may have been resected with the diagnostic biopsy, it suggests that lapatinib is effective in this setting. Therefore, other methods to assess biological activity such as gene expression profile, albeit more expensive and time-consuming, may be more informative. Finally, the molecular events leading to significant tumor regression on MRI were essentially not captured by this study. This may require a larger study and the use of more sensitive and comprehensive molecular tests.

The fundamental question now, having documented drug safety and molecular effects, is what to do next. Certainly, a randomized clinical trial comparing lapatinib versus no treatment in *HER2*-amplified poor-prognosis DCIS with local failure, as the primary endpoint would be required before this treatment can be recommended. Because of the excellent prognosis, the study would include a large number of patients, with a long follow up. Because lapatinib is associated with tumor regression in this setting, a trial assessing whether preoperative administration of the agent results in tumor down-staging, increasing the proportion of patients who can have a breast-sparing procedure, would also be interesting.

To our knowledge, this is the first study evaluating the role of neoadjuvant lapatinib in DCIS. Despite the short half-life of lapatinib (24 hours), its molecular effect in tumoral cells was observed beyond the expected time (4-week period between the last dose of lapatinib and the time of surgery). This phenomenon would indicate that the molecular changes induced by lapatinib are maintained over time. However, this hypothesis should be confirmed with further research.

## Conclusions

The present study shows the feasibility of a short-window treatment with a targeted agent in patients with DCIS. The results demonstrate that standard-dose lapatinib results in signaling modulation by decreasing the *RAS/MAPK* signaling pathway in patients with HER2-positive DCIS. Additional studies are needed to determine if this strategy results in improved patient outcome.

## Authors’ original submitted files for images

Below are the links to the authors’ original submitted files for images. Authors’ original file for figure 1 Authors’ original file for figure 2 Authors’ original file for figure 3 Authors’ original file for figure 4

## Abbreviations

ADCC: antibody-dependent cell mediated cytoxicity
AKT: AKT transforming factor
ALT: alanine transaminase
ASCO/CAP: American Society of Clinical Oncology/College of American Pathologists
AST: aspartate transaminase
BIRADS-2: Breast Imaging Reporting and Data System
CTC: common toxicity criteria
DCIS: ductal *in situ* carcinoma
ECOG: Eastern Cooperative Oncology Group
EGFR: epidermal growth factor receptor
ERK: extracellular-regulated kinase
FFPE: formalin-fixed paraffin-embedded
FISH: fluorescence *in situ* hybridization
HER2: human epidermal growth factor receptor 2
LVEF: left ventricular ejection fraction
MAPK: mitogen-activated protein kinase
MRI: magnetic resonance imaging
NCI: National Cancer Institute
NSABP: National Surgical Adjuvant Breast Project
pAKT: phosphor AKT
PCR: polymerase chain reaction
pEGFR: phospho EGFR
pERK: phospho ERK
PI3K: phosphatidylinositol-4,5-biphosphate 3-kinase
PI3KCA: phosphatidylinositol-4,5-biphosphate 3-kinase, catalytic subunit alpha
PTEN: phosphatase and tensin homolog
RAF: rapid accelerated fibrosarcoma
RAS: rat sarcoma
RECIST: response evaluation criteria in solid tumors
TUNEL: terminal deoxynucleotide transferase-mediated deoxyuridine triphosphate-biotin nick-end labelling
UNL: upper normal limit.
