# Supplementary figures and images for: Molecular effects of lapatinib in patients with HER2 positive ductal carcinoma in situ
Source: Breast Cancer Res. 2014 Sep 4;16:R76. doi: 10.1186/bcr3695 (PMC4448559; doi:10.1186/bcr3695)

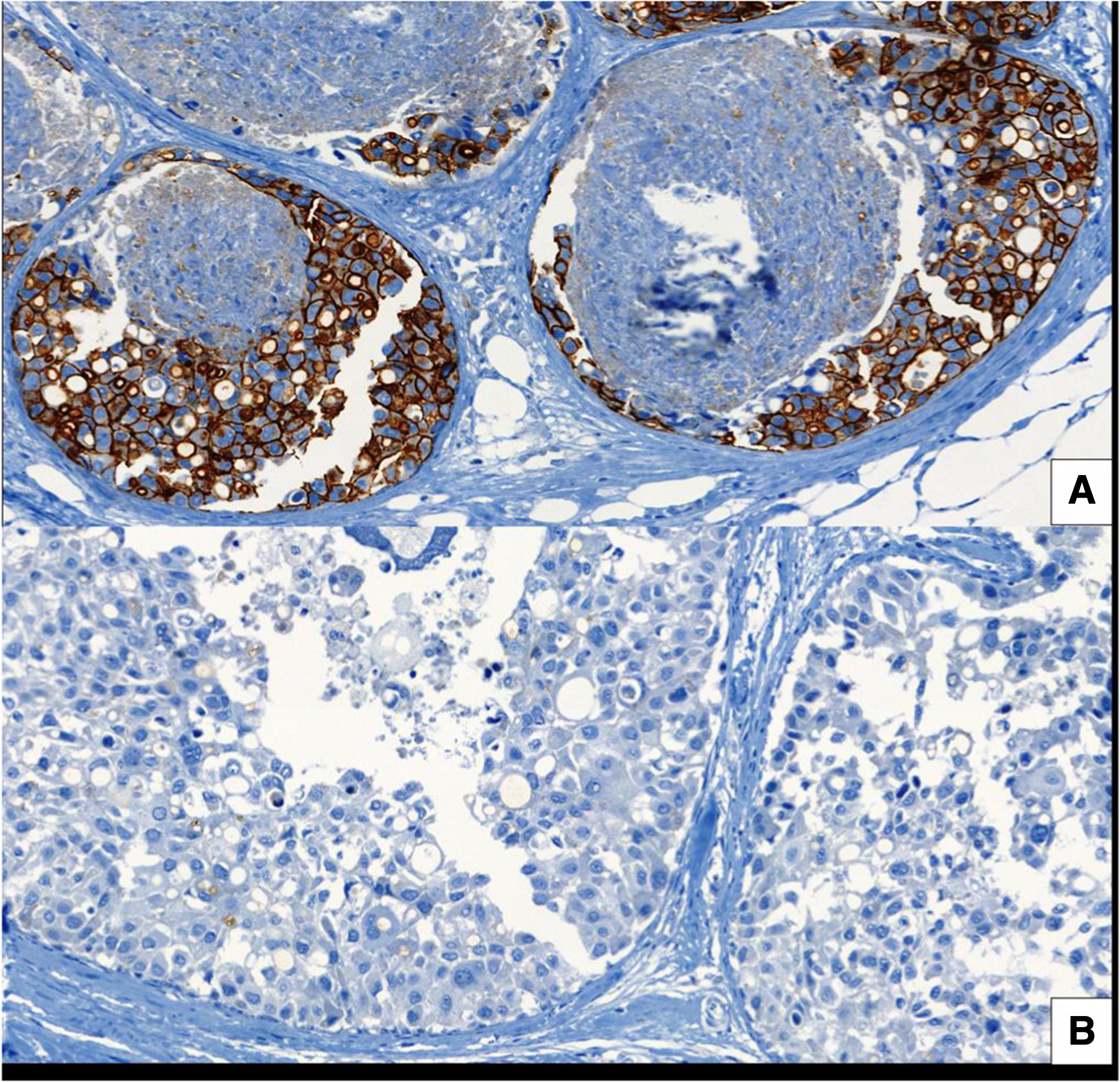

Supplement: Supplementary file 1 — Authors’ original file for figure 1 [file 13058_2013_3564_MOESM1_ESM.tif]

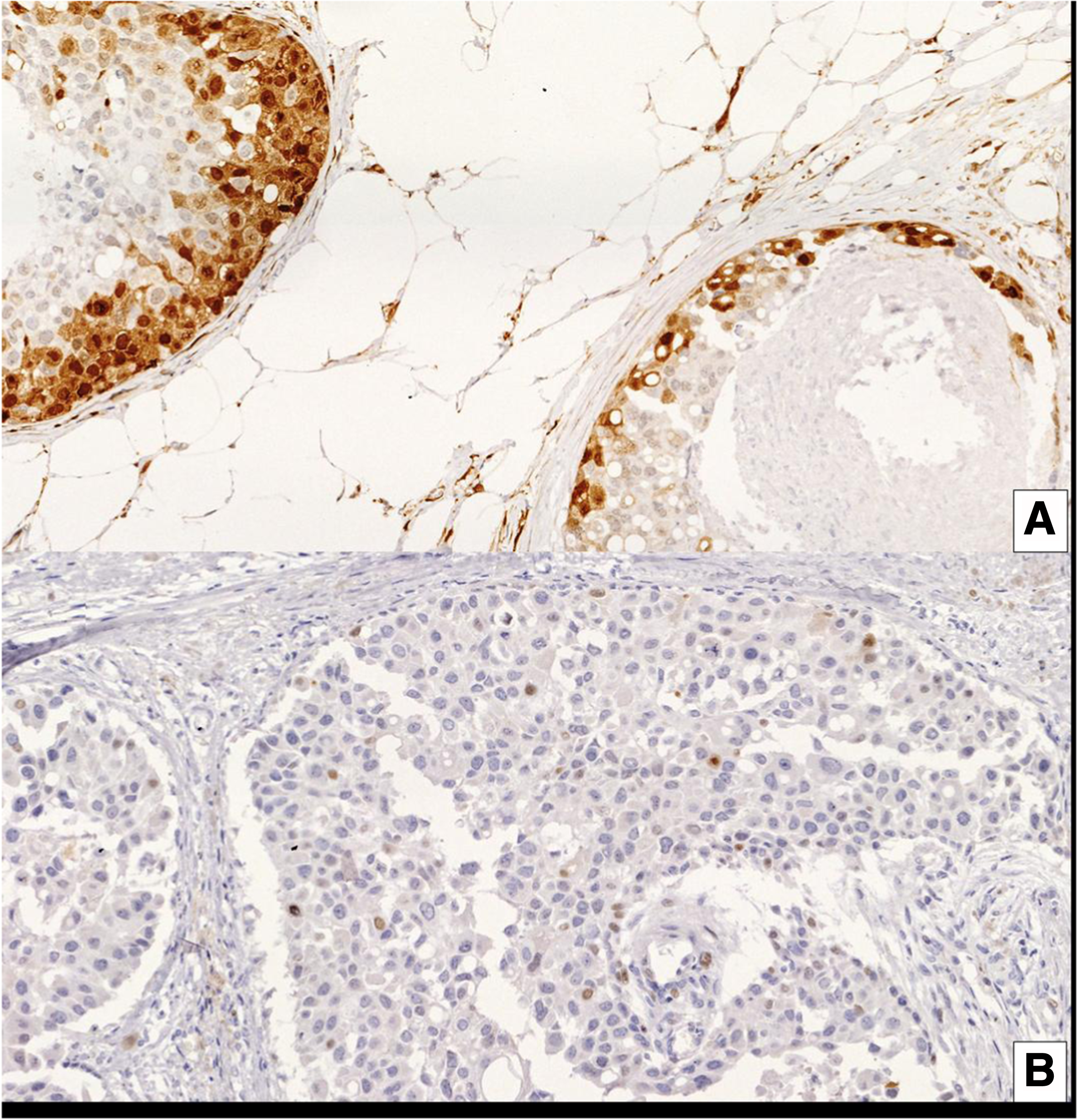

Supplement: Supplementary file 2 — Authors’ original file for figure 2 [file 13058_2013_3564_MOESM2_ESM.tif]

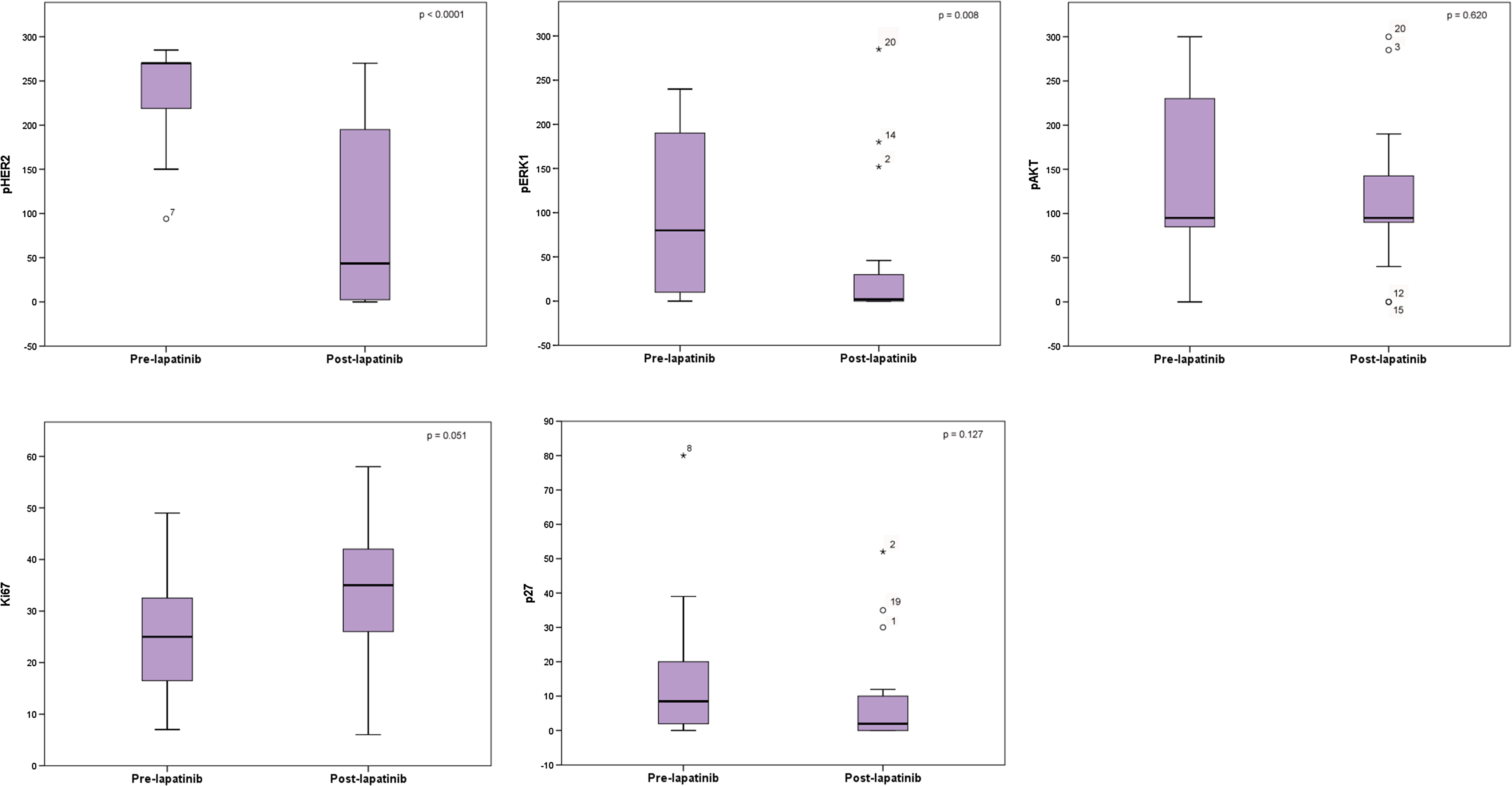

Supplement: Supplementary file 3 — Authors’ original file for figure 3 [file 13058_2013_3564_MOESM3_ESM.tif]

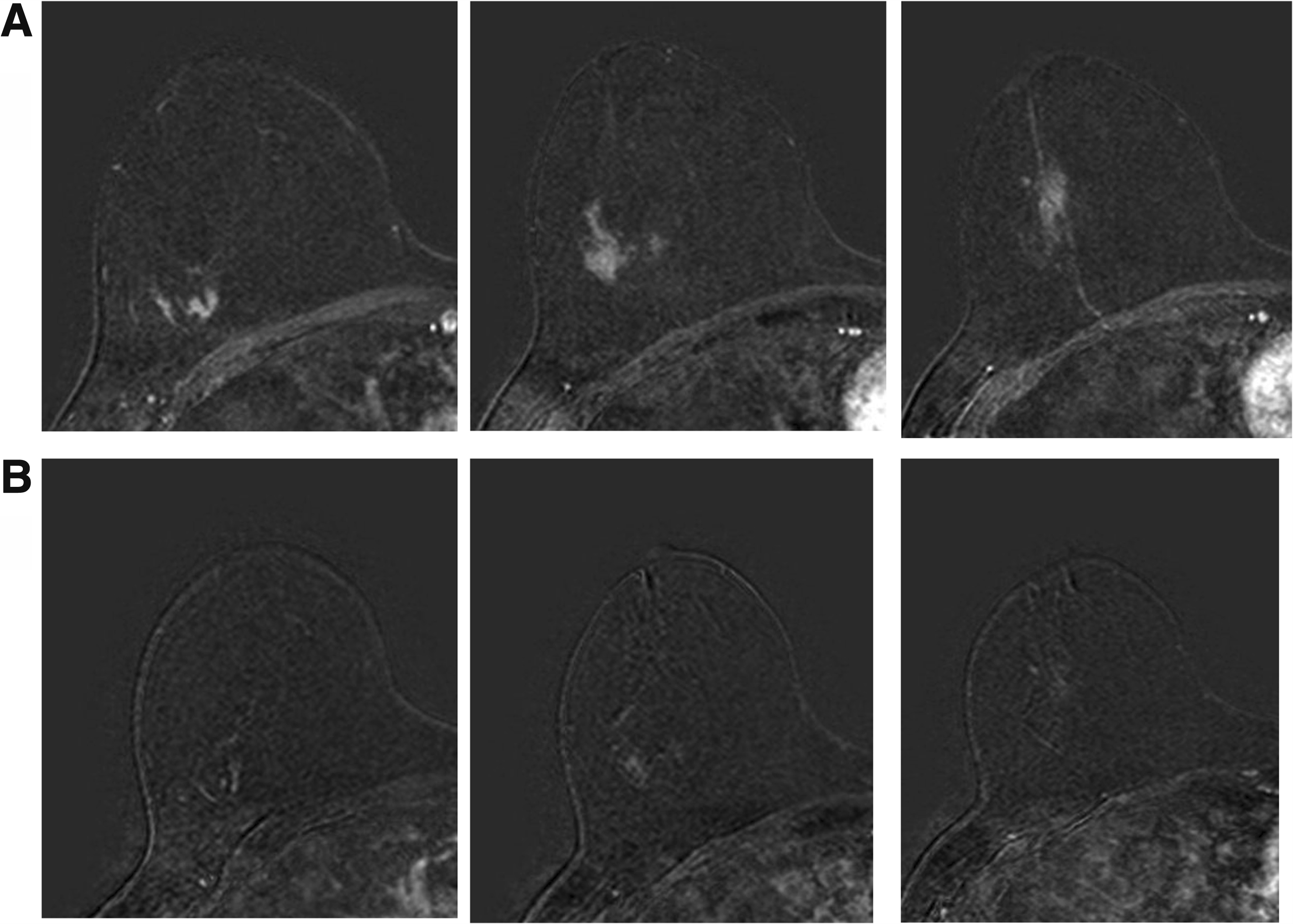

Supplement: Supplementary file 4 — Authors’ original file for figure 4 [file 13058_2013_3564_MOESM4_ESM.tif]
